# Genomic and Phylogenomic Characterization of Three Novel *Corynebacterium* Species from Camels: Insights into Resistome, Mobilome Virulence, and Biochemical Traits

**DOI:** 10.3390/microorganisms13092090

**Published:** 2025-09-08

**Authors:** Haitham Elbir

**Affiliations:** Camel Research Center, King Faisal University, P.O. Box 400, Al-Hasa 31982, Saudi Arabia; helbir@kfu.edu.sa

**Keywords:** dromedary camel, new, species, *Corynebacterium*, genome

## Abstract

The genus *Corynebacterium* is commonly isolated from camel uteri, yet it is rarely identified to the species level. During our routine clinical examination of she-camels brought to the hospital with history of reproductive and systemic health issues, four isolates from the uterus and one isolate from blood could not be assigned to any valid *Corynebacterium* species. Therefore, we aim to identify these isolates, determine any potential virulence factors, and describe how gene turnover contributed to the evolution of these species. Genome-based and phenotypic identification, along with resistome, mobilome, virulome and phylogenomics analysis, was used to characterize the isolates. The isolates were Gram stain-positive, catalase-positive, and rod-shaped. The isolates were assigned to the genus *Corynebacterium* based on 16S rRNA gene sequence similarity and phylogenetic analysis. The isolates 3274 and ayman were classified as two new *Corynebacterium* species based on the average nucleotide identity (ANI) values of 78.46% and 68.88% and digital DNA–DNA hybridization (dDDH) values of 20.9% and 22.4%. The isolates 2581A, 2583C, and 4168A constitute a single *Corynebacterium* species based on their pairwise ANI value of 99% and dDDH value of more than 90%. In addition, isolates 2581A, 2583C, and 4168A showed ANI values of 75.99%, 75.86%, and 76.04% and dDDH values of 23.1%, 23%, and 22.5% with closely related species, and were designated as single new *Corynebacterium* species. Genes for mycolic acid and menaquinone biosynthesis were detected in all isolates. The isolates were susceptible to ceftiofur, linezolid, penicillin, erythromycin, and tetracycline. All isolates harbored the antiseptic resistance gene *qacA*. Moreover, virulence factors involved in cell adhesion and iron acquisition were detected. The evolution of these species is dominated by gene gain rather than gene loss. The majority of these genes are acquired through horizontal gene transfer, mediated by prophages and genomic islands. In summary, we characterized three new *Corynebacterium* species, expanding the number of new *Corynebacterium* species from animals. Moreover, we described the mechanism underlying the genome evolution of these new species. The clinical findings and detection of virulence genes highlight the significance of these isolates as possible pathogens, contributing to the development of endometritis in camels.

## 1. Introduction

The genus *Corynebacterium* currently comprises 166 officially recognized species, as documented by the List of Prokaryotic names with Standing in Nomenclature (https://lpsn.dsmz.de/genus/Corynebacterium accessed on 14 July 2025) [1]. They have mycolic acid-containing cell walls and are Gram-positive straight or slightly curved bacteria. The environmental presence of *Corynebacterium* species is widespread and includes soil, water, and human and animal skin and mucous membranes [2,3,4,5]. The pathogenicity spectrum of *Corynebacterium* includes diseases such as animal mastitis, cattle pyelonephritis, sheep caseous lymphadenitis, and human diphtheria [6,7,8,9,10]. Additionally, *Corynebacterium* was found in the uterus of various animal species [11,12,13,14,15,16,17]. However, few studies have investigated their virulence factors or species-level classification.

*Corynebacterium* species were traditionally classified using biochemical profile and chemotaxonomic investigations, as well as genotypic procedures such as DNA-DNA hybridization and *16S rRNA* gene sequencing [18]. With the advent of genome sequencing technologies and phylogenomic analysis tools, the classification and description of new *Corynebacterium* species increasingly rely on whole-genome sequence analysis [19]. A key method in this process is the average nucleotide identity (ANI) approach and digital DNA–DNA hybridization approach (dDDH), which measure the overall similarity between genome sequences [20,21]. At present, an ANI value in the range of 95–96% is widely accepted as the threshold for delineating bacterial species. Values below this cutoff typically indicate that the organisms belong to new species [22,23,24].

Recently, during our regular identification of bacteria from disease camels, we isolated four *Corynebacterium* species from the uterus of camels with a history of conception failure, as well as one *Corynebacterium* isolate from the blood. Notably, the five isolates listed above were not assigned to a valid *Corynebacterium* based on the current 95–96% ANI cutoff or 70% dDDH cutoff. From an epidemiological standpoint, classification at the species level is critical. Thus, the primary goals of the present study are to (1) utilize genome-based analysis and the biochemical profile to classify the isolated *Corynebacterium* species from camel uterus and blood and (2) clarify the role of gene acquisition and loss on the genome diversity of the isolated *Corynebacterium* species from camel uterus and blood.

## 2. Materials and Methods

### 2.1. Bacterial Isolates

Permission for this study was granted by King Faisal University’s Research Ethics Committee (HAPO-05-HS-003). The study was conducted in the Al-Hasa region of Saudi Arabia at the Camel Research Centre of King Faisal University. During the regular clinical inspection of she-camels brought to the Veterinary Teaching Hospital at King Faisal University, a range of samples were collected and analyzed to investigate potential reproductive and systemic health disease. One particular isolate, designated as ayman, was obtained from the blood of a she-camel suffering from fever. Four isolates designated as 2581C, 2583C, 4168A, and 3274 were isolated from camel uteri with a history of repeated failure to conceive despite multiple breeding attempts. For sample collection, the camel’s skin was disinfected with 70% ethanol before collecting the blood sample from the jugular vein. To ensure aseptic conditions, uterine samples were collected and handled using sterile surgical gloves. The uterine lumen was then sampled with a double-guarded sterile swab (Kruuse, Langeskov, Denmark). The presence or absence of pus in the uterus was further confirmed using transrectal ultrasonography with a 7.5 MHz linear transducer probe (Aloka, Co., Ltd., Tokyo, Japan).

### 2.2. Isolates Genome Sequence Analysis

To extract genomic DNA and form the blood and uterine isolates, the wizard genomic DNA purification kit (Promega Biotech AB, Madison, WI, USA) was utilized following the guidelines provided by the manufacturer. Utilizing the TruSeq Nano DNA Library Preparation Kits (Illumina, San Diego, CA, USA), DNA libraries were constructed for genome sequencing. Using the NovaSeq technology (Illumina) at Macrogen Inc. in Seoul, Republic of Korea, the produced library samples were pooled and put through high-throughput sequencing, yielding paired-end reads of 151 base pairs. After genome sequencing, Fastqc 0.11.8 was used to evaluate the quality of the paired-end reads [25], and Fastp software version 0.20.0 was used to trim low-quality bases [26]. The paired-end reads were de novo assembled using SPAdes 3.15.4 [27]. Then, only scaffolds having a size more than 500 base pairs and more than 10% coverage were kept for analysis.

Prokka 1.14.6 was used for open reading frame prediction, and the Kyoto Encyclopaedia of Genes and Genomes (KEGG) website was used for functional annotation of metabolic KEGG modules, Phosphotransferase System (PTS), and ATP-Binding Cassette (ABC) transporters [28]. In order to identify putative virulence genes, we used the BLAST 2.16.0 program to perform homology searches against the sequence of *Corynebacterium* virulence factors that we retrieved from the virulence factor database (VFDB) [29].

Normally, genomic islands (GIs) contain foreign DNA pieces acquired through horizontal transfer. The IslandViewer 4 web server, which is currently the most accurate method for GI prediction, was used to detect GIs [30]. IslandPick, IslandPath-DIMOB, SIGI-HMM, and Islander are the four genomic island prediction techniques that are integrated into the IslandViewer 4 program. We utilized IslandViewer 4 using default settings. It was generally accepted that the genes identified in the GI were horizontally transmitted genes. Finally, PHASTEST (Phage Search Tool Enhanced Release) (https://phastest.ca, accessed on 10 July 2025) was used to locate prophages in the genomes [31].

### 2.3. Identification of Isolates

Samples were sent to the laboratory and cultured aerobically on 5% sheep blood agar at 37 °C for 48 h. Then, isolates were first screened based on Gram stain. The commercial test API^®^ Coryne system (BioMérieux, Craponne, France) was used to determine the biochemical profile of isolates. The isolates were initially identified as *Corynebacterium* based on a 16S rRNA sequence identity threshold of 98.7% for species delineation, with sequence BLAST comparisons against *Corynebacterium* type species [24]. Finally, the genetic similarity between the blood and uterine isolates and *Corynebacterium* type species was measured using OrthoANI. All pairwise ANI values of the studied species were estimated using the default settings of the command-line version of OrthoANI (OAT v. 1.40 utilizing blastn 2.13.0+) [20]. If two isolates had an OrthoANI value between 95% and 96%, they were regarded as belonging to the same species [24]. The dDDH values were determined using the web tool (http://ggdc.dsmz.de/distcalc2.php, accessed on 20 August 2025) Genome-to-Genome Distance Calculator 3.0 and formula 2 for distance calculations. Isolates were considered a new species if their dDDH values were less than 70% and their OrthoANI values were less than 95–96% [24].

### 2.4. Phylogenomic Analysis

A total of 161 genomes from *Corynebacterium* species with validly published names were downloaded from the GenBank database (https://www.ncbi.nlm.nih.gov/datasets/genome/, accessed on 24 June 2025). All of the downloaded genomes were annotated using PROKKA v1.14.6 in order to retain the same annotation. The GET_HOMOLOGUES scripts v22082022 were used to identify core genes with query coverage of ≥70% and sequence similarity of ≥30% [32]. GET_HOMOLOGUES script analysis yielded 173 single-copy core genes. MAFFT v7.490 was used to align each core gene sequence separately using the default parameters [33]. The software raxmlGUI v 2.0.10, which is a graphical user interface for RaxML v2.0 [34], was used to concatenate the individual MAFFT alignments. For tree reconstruction, the concatenated alignments were used as input for IQ-TREE v3.0.0 [35]. Tree branch support was calculated by 1000 replicates. The resulting tree was visualized using iTOL v7.2.1 [36].

### 2.5. Estimating the Gain and Loss of Genes

Gene family turnover, including gain and loss events, was estimated using BadiRate v1.35, focusing on genomes belonging to the same phylogenetic clades as the *Corynebacterium* isolates. The Gain and Death (GD) model was used to perform BadiRate analysis [37]. A branch-free rates model (GD-FR-ML) was used to calculate the GD rates of gene families under the assumption that each branch has a unique turnover rate. BadiRate analysis requires an ultrametric tree of the taxa studied. We used the ete v 3.1.3 Python library to transform our phylogenomic ML tree into an ultrametric tree [38]. BadiRate analysis additionally requires gene presence and absence data from the GET_HOMOLOGUES script. The resulting tree was visualized using FigTree v1.4.4.

### 2.6. Testing for Antibiotic Susceptibility

Susceptibility to antibiotics was assessed on Mueller–Hinton agar following the Kirby–Bauer disk diffusion method and the European Committee on Antimicrobial Susceptibility Testing, Clinical breakpoints. The sensitivity of isolates to linezolid (30 μg), penicillin (10 μg), erythromycin (15 μg), tetracycline (30 μg), and ceftiofur (30 μg) was examined. The command-line AMRFinderPlus 4.0.3 software [39] was used to detect the existence of resistance determinants. The online Comprehensive Antibiotic Resistance Database (https://card.mcmaster.ca/, accessed on 25 August 2025) was used to detect resistance determinants [40].

## 3. Results

### 3.1. Clinical Data Associated with Isolates

During the regular clinical inspection of she-camels brought to the Veterinary Teaching Hospital at King Faisal University, a range of samples were collected and analyzed to investigate potential reproductive and systemic health issues. One particular isolate, designated as ayman, was obtained from the blood of a she-camel suffering from fever. The blood was free from known blood parasites. The isolates named 2581A, 2583C, 4168A, and 3274 were collected from the uteri of she-camels that presented with vaginal discharge and had a history of repeated failure to conceive despite multiple breeding attempts, and were therefore diagnosed with clinical endometritis.

### 3.2. Phenotypic Classification Characteristics

The five *Corynebacterium* isolates were Gram-positive, rod-shaped bacteria that grew at 37 °C under aerobic conditions on blood agar. The biochemical features of isolates based on API were compared to their closed reference strains, as indicated in Table 1.

### 3.3. Identification Based on 16S rRNA Gene Sequence

**Figure 1:**
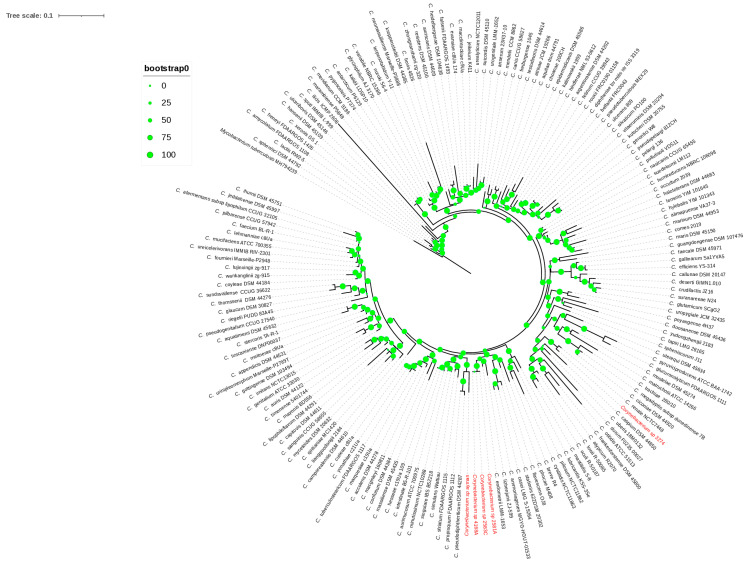
The *16S rRNA* phylogenetic tree. The green circles above the branches are bootstrap support values. Clinical isolates 2581A, 2583C, 4168A, ayman, and 3274 are highlighted in red color.

The *16S rRNA* gene sequences of isolates 4168A, 2581A, and 2583C were 100% similar, whereas isolates 3274 and ayman were 92–94% similar. The isolates 4168A, 2581A, 2583C, 3274, and ayman were identified as *Corynebacterium* species based on the *16S rRNA* gene sequence similarities (Supplementary Table S1). Isolate 3274 clustered with the type species *C. renale* NCTC7448, as shown in the *16S rRNA* phylogeny tree (Figure 1), showing the highest *16S rRNA* gene sequence similarity, 98.95%, with this species. Isolates 4168A, 2581A, and 2583C clustered together with *C. endometrii* LMM-1653, with sequence similarities of 97.41%, 97.50%, and 97.50% (Figure 1). Isolate ayman clustered with the *C. pseudodiphtheriticum* DSM 44287 and *C. propinquum* FDAARGOS 1112 clade but showed the highest *16S rRNA* gene sequence similarity, 95.27%, with *C. humireducens* NBRC 106098 (Supplementary Table S1). The sequence similarity of isolates 4168A, 2581A, 2583C, and ayman was less than the 98.65% cutoff value used to delineate new species. Thus, four putative new species of *Corynebacterium* could be proposed according to the *16S rRNA* gene sequence.

### 3.4. Classification via Genome Sequence

**Figure 2:**
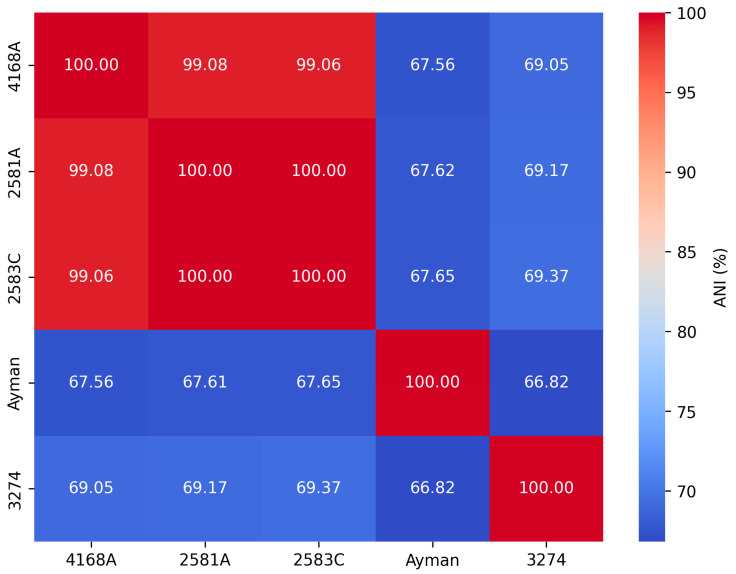
Pairwise ANI values among clinical isolates of *Corynebacterium.*

We calculated ANI values for each clinical isolate against all *Corynebacterium* type species to determine the nearest type species (Supplementary Table S2). The ANI values between the type species *C. endometrii* LMM-1653 and isolates 2581A, 2583C, and 4168A were 75.99%, 75.86%, and 76.04%, while the dDDH values were 23.1%, 23%, and 22.5%, respectively, which were below the cutoff values 95–96% and 70% used to define new species (Figure 2). Thus, they were not classified as *C. endometrii*, but rather as a new *Corynebacterium* species. The pairwise ANI values for isolates 2581A, 2583C, and 4168A were greater than 99%. Thus, they are above the cutoff value 95–96% used to define new species. Also, their dDDH values were greater than 90%, which is above the cutoff 70%, indicating that they belong to the same *Corynebacterium* species. The ANI and dDDH values for isolate 3274 and the type species *C. renale* NCTC7448 were 78.46% and 20.9%, respectively. The ANI and dDDH values of the isolate ayman with the nearest type species *C. pseudodiphtheriticum* DSM 44287 were 68.88% and 22.4%, respectively. The five isolates 2581A, 2583C, 4168A, 3274, and ayman exhibited ANI values lower than the threshold value 95–96% used to define new species, indicating that they represent three distinct *Corynebacterium* species at the genomic level (Figure 2).

**Figure 3:**
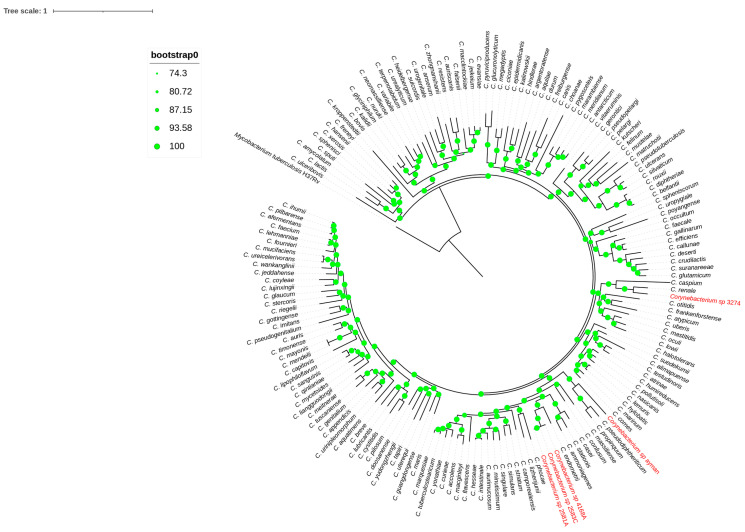
Phylogenomic tree reconstructed from core genes. The green circles above the branches are bootstrap support. Clinical isolates 2581A, 2583C, 4168A, ayman, and 3274 are highlighted in red color.

Core-genome phylogeny inference was the second genome-level method used to group the blood and uterine isolates. A high bootstrap value validated a phylogenomic tree based on 173 single-copy core genes that grouped each isolate to its nearest type species (Figure 3). Remarkably, the phylogenomic topology resembles the 16S rRNA phylogeny tree of the isolates mentioned above. Finally, genes for mycolic acid and menaquinone biosynthesis were detected in all isolates. Phylogenetic and genomic taxonomy analyses showed these isolates as new *Corynebacterium* species, for which we propose the name *Corynebacterium cameli* sp. nov for the isolates 2581A, 2583C, and 4168A, the name *Corynebacterium utericameli* sp. nov for the isolate 3274, and *Corynebacterium hassacameli* sp. nov for the isolate ayman.

### 3.5. Genome Characteristics and Gene Dynamics

The main genome characteristics of the blood and uterine isolates are presented in (Table 2). In summary, the coding densities for isolates 2581A, 2583C, 4168A, ayman, and 3274 were 87.23, 87.33, 87.54% 88.51%, and 90.03%, respectively. Isolates 2581A, 2583C, 4168A, ayman, and 3274 all exhibited similar GC contents of 59.68%, 59.71%, 59.68%, 51.67%, and 58.78%, respectively. Isolates 2581A, 2583C, and 4168A, which belong to same *Corynebacterium* species, share a core genome of 2399 genes. Within this group, the number of strain-specific genes was 120, 13, and 392 for isolates 2581A, 2583C, and 4168A, respectively. In contrast, isolates ayman and 3274, which represent different *Corynebacterium* species, showed much lower gene content similarity with others. Across all five isolates, only 1042 core genes were shared. We detected several different mobile genetic elements, comprising prophages and genomic islands (Table 3). In brief, one intact prophage region with the best match to PHAGE_Coryne_Juicebox NC_048070 was detected across the genome of isolates 2483C, 2581A, 4168A, and 3274. Furthermore, isolate 4168A and 3274 carried one intact prophage region with the best match to PHAGE_Gordon_Nyceirae_NC_031004 and PHAGE_Rhodoc_Hiro_NC_048669, respectively.

Additionally, we found that 12.9%, 9.05%, and 12.58% of isolate genes exist within a pool of genomic islands in isolates 2581A, 2583C, and 4168A, respectively. In the case of isolate 3274 and ayman, we found that 6.18% and 6.81% of isolate genes exist within a pool of genomic islands. These genes are involved in metabolism, genetic information processing, environmental information processing, and cellular processes.

**Figure 4:**
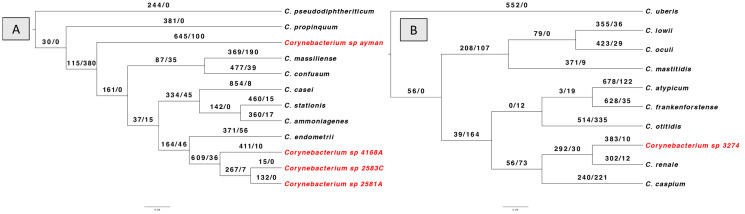
BadiRate analysis results according to branch-free rates model (GD-FR-ML). The numbers on the branches indicate gains gain/gene loss number. The clinical *Corynebacterium* isolates are highlighted in red color. (**A**) shows gene turnover for isolates 2581A, 2583C, 4168A, and ayman. (**B**) shows the gene turnover for isolate 3274.

Lastly, using the BadiRate v1.35 software, we looked into gene gain/loss (turnover) events for every *Corynebacterium* isolate through the phylogeny in order to further analyze the dynamics of gene content variation (Figure 4). The results revealed that the number of genes gained outnumbers the number of genes lost in all isolates. Gene gain events were highest in the clade that included isolates 2581A, 2583C, and 4168A. In contrast, the clade containing isolates 3274 had the fewest gene gain events. Furthermore, the turnover rate for gene gain and loss is comparable between isolate 3274 and its nearest type species *C. renale.*

### 3.6. Analysis of Functional Characteristics

**Figure 5:**
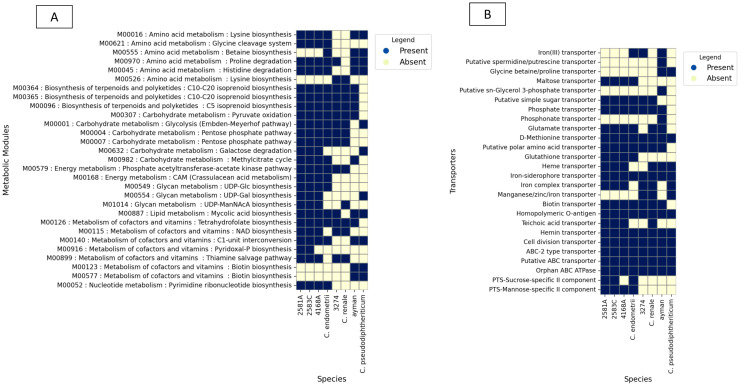
KEGG BLAST analysis results for isolates 2581A, 2583C, 4168A, 3274, and ayman and their type species. (**A**) shows metabolic modules. (**B**) shows ABC and PTS transporters.

To examine the functional profile of *Corynebacterium* isolates, the transporters and metabolic modules were assembled for every genome using the KEGG database. A detailed examination of metabolic potentials reveals that uterine isolates 2581A, 2583C, and 4168A encode for 63, 63, and 62 complete KEGG metabolic modules and 20, 20, and 19 transporters, respectively. As for the difference from its nearest type species *C. endometrii* from the cow uterus, it encodes 58 complete KEGG metabolic modules and 18 transporters (Figure 5).

In the case of the isolate 3274, examination of metabolic potentials reveals that it encodes 52 complete KEGG metabolic modules and 15 transporters. As for the difference from it its nearest type species *C. renale* (cow isolate), it encodes 51 complete KEGG metabolic modules and 17 transporters (Figure 5).

In the case of the blood isolate ayman, examination of metabolic potentials reveals that it encodes 52 complete KEGG metabolic modules and 18 transporters. As for the difference from it its nearest type species *C. pseudodiphtheriticum*, it encodes 49 complete KEGG metabolic modules and 11 transporters (Figure 5).

### 3.7. Virulence Genes

Analysis of the five *Corynebacterium* genomes against the VFDB revealed genes involved in adherence. These include genes for the *SpaA* and *SpaD* of pili and DIP_RS14950 (Supplementary Table S3). The diphtheria toxins and *pld* genes were not detected in the five *Corynebacterium* isolates, but the *dtxR* regulator gene was present in the five *Corynebacterium* isolates. Notably, the post-translational modification gene DIP_RS20575, encoding an *MdbA* thioredoxin-like protein, was present in all isolates. Iron transporters were widely distributed in the isolates, including the heme transporter encoded by *hmuTUV* genes, the iron (III) transporter encoded by *AFuABC* genes, and the iron siderophore ABC transporter encoded by *FepBDGC* and *CeuABCD* genes (Supplementary Table S3). Genome mining revealed that all isolates shared three out of four two-component systems (TCS): (1) *SenX3*-*RegX3*, which has roles in the phosphate starvation response, (2) *MprAB*, which is essential in the maintenance of persistent infection, and (3) *MtrAB*, which has roles in the osmotic stress response. The fourth TCS, *DesK-DesR*, which regulates membrane lipid fluidity, is only found in isolates 4168A and ayman.

### 3.8. Antibiotic Susceptibility Profile

The uterine isolates and blood isolates showed similar phenotypic antibiotic susceptibility as determined by the disk diffusion method. All the isolates were sensitive to linezolid, penicillin, erythromycin, tetracycline, and ceftiofur. Then, we analyzed the resistomes of uterine isolates and blood isolates. No resistance determinants were found using the command-line AMRFinderPlus 4.0.3 software and by blast against the Comprehensive Antibiotic Resistance Database. KEGG annotation revealed the existence of the multidrug efflux pump encoded by the *qacA* gene in all isolates, and this was further confirmed by blast against the Comprehensive Antibiotic Resistance Database.

## 4. Discussion

Here, during our routine clinical examination of the uteri of she-camels with a history of infertility, we found three distinct novel *Corynebacterium* species. Currently, species identification follows a three-step approach: initial classification based on *16S rRNA* gene sequence similarity, followed by phylogeny inference and calculation of ANI or dDDH value [24]. The *16S rRNA* gene has been a rapid and accurate genetic method for bacterial identification for many years [41]. Nevertheless, because of its low resolution, this marker is unable to accurately classify some bacteria to the species level [42,43]. The pairwise similarity values of the *16S rRNA* gene for isolate 3274 versus validly published *Corynebacterium* species in this study demonstrated the limits of this gene analysis. According to BLAST results, the similarity was higher than the usual threshold of 98.7% for species delineation [44]. Consequently, isolate 3274 was assigned to *C. renale*. In contrast, isolate 3274 was considered a new *Corynebacterium* species, as its dDDH value was less than 70% and its OrthoANI value was less than 95–96% [23]. Thus, this indicates the possibility of incorrect classification when depending exclusively on *16S rRNA* gene sequences. Therefore, all isolates were subjected to further genetic testing, including dDDH, OrthoANI, and phylogeny, to achieve correct classification. Finally, to elucidate the evolutionary relationships, taxonomists currently mostly rely on phylogenetic tree inference [45]. Notably, single-gene phylogenetic inference usually provides less resolution than phylogenomic methods, which combine information from across the genome. Consequently, a phylogenomic tree was inferred based on 173 single-copy core genes. Each isolate clustered with its closest type strain, providing further support for their taxonomic classification of the isolates into three distinct novel *Corynebacterium* species.

Second, for each isolate, we mapped gene gain and loss events in the *Corynebacterium* phylogeny to gain a better understanding of the dynamics of gene content variations. We observed a dynamic pattern of genome evolution characterized by a higher rate of gene gain than loss on the terminal branch. Our results align with previous research on the human pathogen *Acinetobacter baumannii*, in which gene gain was a feature of evolution in the terminal branch [46]. Therefore, gene turnover contributed to the overall diversity of gene repertoires of blood and uterine isolates. The predominance of gene gain over gene loss might indicate that these species are expanding or altering their niche. We found various types of mobile genetic elements, consisting of prophages and genomic islands, implying that genetic transfer occurs frequently in *Corynebacterium* isolates from blood and uterine samples. Therefore, the notable gene gain found in the clinical isolates can be attributed to the horizontal gene transfer mediated by mobile genetic elements. The functional implications of these horizontally transferred genes contributed to genetic diversity among the isolates. In addition, this resulted in enhanced metabolism by gaining genes involved in carbohydrate and amino acid metabolism, as well as transporters. The uterine isolates showed different metabolic profiles although they shared the same niche. Their metabolic profile likely represents their previous niche.

The identification of the *MprAB*, *SenX3*-*RegX3*, and *MtrAB* two-component systems, associated with osmotic stress response and persistent infection in the human pathogen *Mycobacterium tuberculosis* [47,48], suggests that our clinical isolates have a gene set supporting their survival in the animal host. Additionally, iron acquisition system detection, which allows sequestered iron to be extracted from the host, suggests adaptation to the iron-limited conditions commonly found in host habitats. In addition, the metabolic modules identified in our clinical isolates support core survival functions and basic metabolism rather than pathways associated with the degradation of environmental contaminants such as aromatic compounds and benzoate derivatives. Clinical findings, along with the above observations, suggest that our isolates are better adapted to survival in the animal host environment and are therefore more likely to represent potential animal pathogens rather than environmental contaminant bacteria.

The detection of *Corynebacterium diphtheriae SpaA-* and *SpaD*-type pili in our clinical isolates, which is implicated in host cell attachment, suggests that our clinical isolates have the capacity for colonization [49]. This is further supported by the presence of virulence factor DIP0733, which has been implicated in both the adhesion and invasion of host cells. Moreover, the blood isolate ayman harbored two complete biotin biosynthesis modules (M00123 and M00577), suggesting a self-sufficient metabolic capability that may enhance survival in biotin-limited environments, an attribute linked to increased virulence in pathogens such as *Mycobacterium tuberculosis* [50]. In contrast, the uterine isolates lacked these biosynthetic modules but contained a biotin uptake transporter system, implying a greater dependence on the host-derived biotin. Collectively, these genomic features, including adhesion and invasion factors and metabolic adaptations homologous to those in *Corynebacterium diphtheriae*, suggest that the isolates have pathogenic potential and may pose a risk for human infection. Further functional studies are warranted to clarify their role in disease and assess their clinical significance.

The resistome profile and the antimicrobial phenotypic results agreed, and all isolates showed sensitivity to the tested drugs. These findings hold significant practical implications for the treatment of endometritis, where ceftiofur is commonly used as the primary therapeutic agent [51]. However, the effectiveness of standard sanitization methods in veterinary reproductive control may be hampered by the identification of antiseptic resistance genes *qacA*, which have been shown to mediate antiseptic resistance in *Staphylococcus aureus* [52]. As a result, resistant bacteria may persist in agricultural or clinical settings, which may pose a risk for zoonotic infection for humans in close contact with camels.

In conclusion, through genome-based taxonomy, we identified three new *Corynebacterium* species out of five isolates, increasing the total number of *Corynebacterium* species isolated from animals. Species evolution is dominated by gene gain over gene loss. The majority of the gained genes were acquired through horizontal gene transfer assisted by genomic islands and prophages. The existence of several virulence genes in these species and their isolation from camel with endometritis and fever may suggest that these species are pathogenic. Nevertheless, more proof is required to demonstrate their pathogenicity.

## Figures and Tables

**Table 1:** Differential features of isolates 2581A, 2581A, and 4168A and the type strain of the closest related species *C. endometrii*. The isolate ayman and the type strain of the closest related species *C. pseudodiphtheriticum*. d stands for variable.

Features	2581A	2581A	4168A	*C. endometrii*	Ayman	*C. pseudodiphtheriticum*	3274	*C. renale*
Nitrate reduction	-	-	-	-	-	+	-	-
Pyrazinamidase	+	+	+	+	+	+	+	+
Pyrrolidonyl arylamidase	+	+	+	-	-	d	-	-
Alkaline phosphatase	-	-	-	-	+	d	-	-
β-Glucuronidase	-	-	-	-	-	-	-	+
β-Galactosidase	-	-	-	-	-	-	-	-
α-Glucosidase	-	-	-	-	-	-	-	-
N-Acetyl-β-glucosaminidase	-	-	-	-	-	-	-	-
Esculin hydrolysis	-	-	-	-	-	-	-	-
Urease (urea hydrolysis)	-	-	-	-	-	+	+	+
Gelatin hydrolysis	-	-	-	-	-		-	-
Glucose fermentation	+	+	+	+	-	-	+	+
Ribose fermentation	+	+	+	-	-	-	-	+
Xylose fermentation	-	-	-	+	-	-	-	-
Mannitol fermentation	-	-	-	-	-	-	-	-
Maltose fermentation	+	+	+	+	-	-	-	-
Lactose fermentation	-	-	-	-	-	-	-	-
Sucrose fermentation	-	-	-	-	-	-	-	-
Glycogen fermentation	-	-	-	-	-	-	-	_
Catalase	+	+	+	+	+	+	+	+

**Table 2:** General genomic characteristics of *Corynebacterium* clinical isolates.

Species	Size (bp)	CDS	rRNA	tRNA	tmRNA	CRISPR	Accession Number
*Corynebacterium* sp. 2581A	2,990,088	2829	3	54	1	-	SAMN49893244
*Corynebacterium* sp. 2583C	2,863,892	2718	3	54	1	-	SAMN49893245
*Corynebacterium* sp. 4168A	2,965,271	2846	4	54	1	-	SAMN49893247
*Corynebacterium* sp. 3274	2,378,403	2249	3	54	1	1	SAMN49893246
*Corynebacterium* sp. ayman	2,305,594	2114	3	53	1	1	SAMN49893248

**Table 3:** Types and features of mobile genetic elements detected in the genomes of the studied isolates.

Species	Prophage			Genomic Island		
	Size (Kb)	Total Genes	Number	Total Size (bp)	Total Genes	Number
*Corynebacterium* sp. 2581A	46.7	71	1	334,281	365	26
*Corynebacterium* sp. 2583C	46.7	71	1	223,521	246	17
*Corynebacterium* sp. 4168A	44.4, 39.3	67, 44	2	311,761	358	25
*Corynebacterium* sp. 3274	41, 22.8	53, 26	2	134,505	139	11
*Corynebacterium* sp. ayman	-	-	-	119,830	144	6

## Data Availability

Data are available on a publicly accessible GenBank website, and the accession number is mentioned in Table 2. Further inquiries can be directed to the corresponding author.

## Supplementary Materials

The following supporting information can be downloaded at https://www.mdpi.com/article/10.3390/microorganisms13092090/s1, Table S1: Values for 16S rRNA, from pairwise comparison of *Corynebacterium* type species against clinical *Corynebacterium* isolates. Table S2: Values for OrthoANI, from pairwise comparison of *Corynebacterium* type species against clinical *Corynebacterium* isolates; Table S3: Virulence factors genes of *Corynebacterium* isolates from camel uterus and blood.
