# Supplementary material for: Genomic and Phylogenomic Characterization of Three Novel Corynebacterium Species from Camels: Insights into Resistome, Mobilome Virulence, and Biochemical Traits
Source: Microorganisms. 2025 Sep 8;13(9):2090. doi: 10.3390/microorganisms13092090 (PMC12472555; doi:10.3390/microorganisms13092090)
Supplement: Supplementary file 1 [file microorganisms-13-02090-s001.zip › microorganisms-3870504-supplementary.pdf]

Supplementary table S1. Values for 16S rRNA, form pairwise comparison of *Corynebacterium* type species against clinical *Corynebacterium* isolates. For each isolate, best match was highlighted in yellow colour.

Species	2581A	2583C	4168A	3274	ayman
C. hansenii DSM 45109	94.61	94.61	94.41	93.61	94.42
C. xerosis GS 1	94.67	94.67	94.48	93.47	94.35
C. freneyi FDAARGOS 1426	95.07	95.07	94.89	93.83	94.22
C. sphenisci DSM 44792	94.61	94.61	94.41	94.21	93.70
C. lactis RW2-5	94.93	94.93	94.75	93.62	93.89
C. amycolatum FDAARGOS 1108	94.79	94.79	94.60	93.24	93.64
C. sputi IMMIB	94.13	94.13	93.91	92.63	93.73
C. humireducens NBRC 106098	95.92	95.92	95.77	94.89	95.27
C. ulceribovis DSM 45146	94.40	94.40	94.20	92.27	93.50
C. pollutisoli VDS11	96.18	96.18	96.04	95.64	95.20
C. suedekumii	96.19	96.19	96.05	95.18	95.21
C. marambiense P5848	94.55	94.55	94.35	93.70	93.67
C. kalidii LD5P10	96.43	96.43	96.30	93.91	94.23
C. pygosceleis P7374	94.42	94.42	94.22	93.47	93.40
C. nasicanis	95.99	95.99	95.83	95.64	94.69
C. variabile NBRC 15286	96.31	96.31	96.17	94.22	94.16
C. antarcticum P6129	94.48	94.48	94.29	93.70	93.60
C. neomassilliense	96.11	96.11	95.97	94.37	94.30
C. glyciniphilum AJ 3170	95.61	95.61	95.45	92.93	93.82
C. terpenotabidum Y-11	95.74	95.74	95.59	93.92	93.81
C. meridianum CCM 9186	94.49	94.49	94.29	93.78	93.60
C. nuruki S6	95.07	95.07	94.89	93.99	93.91
C. bovis 4826	94.80	94.80	94.61	93.37	93.51
C. halotolerans DSM 44683	94.94	94.94	94.76	94.23	93.78
C. hylobatis	95.92	95.92	95.77	94.29	94.42
C. aquilae dsm 44791	95.81	95.81	95.66	95.07	93.91
C. spheniscorum J11	95.47	95.47	95.29	95.50	93.67
C. lemuris	95.85	95.85	95.70	93.91	94.23
C. tapiri LMG 28165	95.15	95.15	94.98	95.05	93.91
C. endometrii LMM-1653	97.50	97.50	97.41	94.81	94.29
C. evansiae c8Ua	95.97	95.97	95.97	95.80	94.60
C. zhongnanshanii zg	95.47	95.47	95.30	93.62	93.52
C. pelargi 136	94.90	94.90	94.71	96.10	94.56
C. pseudopelargi 812CH	94.95	94.95	94.77	96.17	94.49
C. rouxii FRC0190	95.66	95.66	95.51	95.65	93.77

C. marinum DSM 44953	95.32	95.32	95.15	94.14	94.17
C. felinum CCUG 39943	96.16	96.16	95.98	94.79	93.23
C. macclintockiae c9Ua	95.73	95.73	95.58	93.48	93.97
C. camporealensis DSM 44610	95.60	95.60	95.44	94.74	94.42
C. diphtheriae bv mitis str ISS 3319	95.20	95.20	95.02	95.72	93.46
C. occultum 2039	95.46	95.46	95.29	94.52	93.64
C. falsenii FDAARGOS 1493	95.66	95.66	95.50	93.94	93.39
C. resistens DSM 45100	94.87	94.87	94.68	93.76	93.12
C. uropygiale JCM 32435	95.37	95.37	95.20	95.00	93.67
C. jeikeium K411	95.54	95.54	95.38	93.41	93.72
C. gerontici W8	95.27	95.27	95.10	96.02	93.97
C. belfantii FRC0043	95.52	95.52	95.36	95.87	93.65
C. vitaeruminis DSM 20294	95.66	95.66	95.50	95.65	93.72
C. stercoris	94.94	94.94	94.67	94.68	93.93
C. hindlerae NML 93-0612	95.92	95.92	95.77	94.37	94.09
C. gallinarum	95.07	95.07	94.89	94.58	93.32
C. efficiens YS-314	94.94	94.94	94.76	94.58	93.25
C. choanae 200CH	95.53	95.53	95.37	95.12	93.32
C. lizhenjunii ZJ-599	96.45	96.45	96.32	94.15	94.63
C. aquatimens DSM 45632	95.46	95.46	95.29	94.51	93.96
C. coyleae DSM 44184	94.56	94.56	94.37	95.12	93.44
C. confusum DSM 44384	95.71	95.71	95.56	94.21	94.41
C. uberis 18M0132	94.44	94.44	94.24	96.02	93.01
C. alimapuense VA37	95.34	95.34	95.17	94.29	93.91
C. guangdongense DSM 107476	94.08	94.08	93.87	93.63	94.09
C. epidermidicanis DSM 45586	95.60	95.60	95.44	95.57	93.84
C. faecale DSM 45971	95.60	95.60	95.44	94.43	93.45
C. freiburgense 1045	95.01	95.01	94.90	94.59	93.20
C. suranareeae N24	94.50	94.50	94.30	95.04	92.81
C. urogenitale LMM-1652	94.56	94.56	94.37	94.09	93.53
C. glutamicum SCgG2	94.25	94.25	94.04	95.11	92.75
C. urinipleomorphum Marseille-P2799T	96.00	96.00	95.85	94.97	94.00
C. casei LMG S-19264	95.21	95.21	95.04	94.00	94.02
C. argentoratense DSM 44202	96.32	96.32	96.25	94.74	93.97
C. urealyticum NCTC12011	95.46	95.46	95.29	94.44	93.57
C. simulans Wattiau	97.36	97.36	97.27	94.21	94.87
C. pilbarense CCUG 57942	94.77	94.77	94.50	95.34	92.80
C. renale NCTC7448	94.81	94.81	94.63	98.95	93.38
C. auriscanis DSM 44609	94.93	94.93	94.75	93.62	92.86
C. appendicis DSM 44531	95.91	95.91	95.76	94.51	94.41

C. flavescens OJ8	96.20	96.20	96.07	94.18	94.25
C. kroppenstedtii DSM 44385	93.62	93.62	93.39	92.16	93.25
C. faecium	94.56	94.56	94.37	94.82	93.31
C. suicordis DSM 45110	93.40	93.40	93.16	93.40	92.69
C. doosanense DSM 45436	93.86	93.86	93.64	94.75	92.42
C. comes 2019	94.62	94.62	94.43	94.07	93.73
C. ulcerans 809	95.01	95.01	94.83	96.77	93.39
C. fournieri Marseille-P2948	94.89	94.89	94.71	94.97	93.63
C. auris DSM 44122	95.00	95.00	94.82	94.38	93.76
C. anserum 23H37	94.88	94.88	94.69	94.30	93.45
C. riegelii PUDD 83A45	94.81	94.81	94.62	94.29	93.25
C. atrinae JCM 19266	95.31	95.31	95.14	95.00	93.06
C. testudinoris DSM 44614	95.62	95.62	95.46	94.99	93.22
C. mastitidis S-8	94.13	94.13	93.99	95.12	93.39
C. callunae DSM 20147	94.57	94.57	94.37	94.74	92.82
C. stationis 622DSM 20302	95.15	95.15	94.97	93.92	93.77
C. meitnerae c8Ua 172	97.12	97.12	97.01	95.34	93.92
C. crudilactis JZ16	94.43	94.43	94.23	94.58	92.88
C. pseudotuberculosis MEX29	94.75	94.75	94.56	96.62	93.25
C. lipophiloflavum DSM 44291	95.53	95.53	95.37	95.04	93.90
C. qintianiae MC1420	95.66	95.66	95.50	95.64	93.45
C. tuberculostearicum FDAARGOS 1117	95.85	95.85	95.84	94.67	95.14
C. glaucum DSM 30827	94.30	94.30	94.09	94.15	93.18
C. sanguinis CCUG 58655	95.08	95.08	94.90	95.05	93.71
C. striatum FDAARGOS 1115	97.10	97.10	96.99	93.91	94.48
C. maris DSM 45190	93.70	93.70	93.48	93.76	92.92
C. liangguodongii 2184	95.45	95.45	95.29	95.41	93.83
C. poyangense 4H37	95.26	95.26	95.09	95.20	93.71
C. ureicelerivorans IMMIB RIV	94.56	94.56	94.37	95.35	93.37
C. genitalium ATCC 33030	95.78	95.78	95.63	95.20	94.02
C. silvaticum PO100	95.01	95.01	94.83	96.62	93.25
C. phocae M408 89	95.92	95.92	95.78	93.55	94.35
C. deserti GIMN1.010	94.23	94.23	94.03	95.41	92.46
C. ciconiae DSM 44920	93.81	93.81	93.53	93.49	93.65
C. yonathiae c21Ua	96.24	96.24	96.24	94.67	94.81
C. curieae c8Ua 181	96.24	96.24	96.24	94.67	94.81
C. oculi R-50187	93.78	93.78	93.60	95.31	92.90
C. mucifaciens ATCC 700355	94.56	94.56	94.37	95.42	92.99
C. tuscaniense DNF00037	96.50	96.50	96.38	95.27	93.95
C. marquesiae c19Ua 121	96.17	96.17	96.17	94.82	94.94

C. lujinxingii zg-917	95.01	95.01	94.83	94.59	93.89
C. kalinowskii 1959	95.66	95.66	95.50	95.05	93.78
C. aurimucosum ATCC 700975	96.96	96.96	96.99	94.44	94.73
C. canis CCUG 58627	94.70	94.70	94.70	95.08	92.64
C. mendelii	95.14	95.14	94.97	95.05	93.64
C. mayonis	95.14	95.14	94.97	95.05	93.64
C. ammoniagenes MGYG-HGUT-01533	94.70	94.70	94.50	93.70	93.44
C. gottingense DSM 103494	94.93	94.93	94.75	95.42	93.82
C. minutissimum NCTC10288	97.10	97.10	97.13	94.52	94.74
C. singulare IBS B52218	96.77	96.77	96.79	94.44	94.60
C. mycetoides DSM 20632	94.94	94.94	94.76	95.57	93.36
C. breve R4	95.31	95.31	95.14	94.88	92.66
C. lubricantis KSS-3Se	94.69	94.69	94.69	95.44	93.46
C. lehmanniae c8Ua 144	95.12	95.12	94.93	94.81	92.69
C. intestinale New 00970	96.91	96.91	96.94	94.44	94.62
C. hesseae c19Ua 109	96.91	96.91	96.94	94.44	94.62
C. afermentans subsp lipophilum CCUG 32105	94.76	94.76	94.57	95.20	93.77
C. caspium DSM 44850	93.45	93.45	93.15	93.90	92.81
C. wankanglinii zg-915	95.04	95.04	94.85	94.44	93.44
C. macginleyi 160811	96.44	96.44	96.44	94.52	94.35
C. imitans NCTC13015	95.06	95.06	94.88	95.42	93.37
C. timonense 5401744	95.85	95.85	95.70	94.82	93.22
C. kutscheri DSM 20755	94.32	94.32	94.12	95.73	93.08
C. massiliense DSM 45435	94.42	94.42	94.22	94.47	94.16
C. capitovis DSM 44611	94.56	94.56	94.37	94.67	92.80
C. jeddahense DSM 45997	95.26	95.26	95.09	94.37	93.30
C. pilosum NCTC11862	95.26	95.26	95.09	94.67	93.82
C. lowii R-50085	93.81	93.81	93.64	94.59	93.22
C. accolens DSM 44278	96.31	96.31	96.31	94.83	94.35
C. pseudodiphtheriticum DSM 44287	94.80	94.80	94.61	93.33	95.01
C. propinquum FDAARGOS 1112	94.61	94.61	94.41	93.47	95.13
C. ihumii	94.88	94.88	94.70	95.27	93.63
C. mustelae DSM 45274	94.58	94.58	94.38	95.51	93.08
C. pseudogenitalium CCUG 27540	94.35	94.35	94.15	95.04	92.40
C. uterequi DSM 45634	93.53	93.53	93.29	94.73	92.82
C. frankenforstense DSM 45800	92.28	92.28	92.00	94.76	91.89
C. glucuronolyticum FDAARGOS 1111	92.98	92.98	92.73	93.84	92.13
C. megadyptis subsp dunedinense7B 00735	93.59	93.59	93.36	94.04	93.12
C. yudongzhengii 2183	93.26	93.26	93.02	93.87	93.12
C. atypicum R2070	92.36	92.36	92.08	93.73	92.42

C. cystitidis NCTC11863	94.30	94.30	94.09	94.17	92.28
C. heidelbergense DSM 104638	93.39	93.39	93.15	92.47	92.57
C. durum F0235	92.86	92.86	92.86	94.04	92.45
C. pyruviciproducens ATCC BAA-1742	92.73	92.73	92.47	93.76	91.58
C. otitidis ATCC 51513	91.17	91.17	90.86	92.75	90.95
C. matruchotii ATCC 14266	91.89	91.89	92.57	93.68	91.45

Supplementary table S2. Values for OrthoANI, form pairwise comparison of *Corynebacterium* type species against clinical *Corynebacterium* isolates.

**Species**	**2581A**	**2583C**	**4168A**	**3274**	**ayman**
C. endometrii LMM-1653	75.99	75.86	76.04	69.51	67.72
C. aurimucosum ATCC 700975	73.59	73.65	73.75	69.52	67.81
C. hesseae c19Ua 109	73.54	73.46	73.47	69.68	67.68
C. intestinale	73.54	73.44	73.58	69.71	67.64
C. confusum DSM 44384	73.50	73.63	73.67	70.23	67.81
C. singulare IBS B52218	73.47	73.60	73.37	69.53	67.87
C. tuberculostearicum FDAARGOS 1117	73.33	73.12	73.20	69.65	68.23
C. striatum FDAARGOS 1115	73.26	73.29	73.54	69.73	67.74
C. minutissimum NCTC10288	73.10	73.09	73.21	69.45	67.80
C. simulans Wattiau	73.02	73.04	73.36	69.60	67.60
C. marquesiae c19Ua 121	72.95	72.94	72.88	69.38	67.99
C. camporealensis DSM 44610	72.87	72.90	72.92	69.70	68.03
C. accolens DSM 44278	72.85	72.84	72.70	69.62	67.98
C. curieae c8Ua 181	72.56	72.52	72.68	69.35	67.97
C. lizhenjunii ZJ-599	72.49	72.48	72.47	69.18	67.79
C. massiliense DSM 45435	72.46	72.43	72.30	69.69	67.41
C. yonathiae c21Ua 68	72.36	72.40	72.64	69.09	67.95
C. flavescens OJ8	72.27	72.30	72.35	69.08	67.89
C. phocae M408 89 1	71.97	71.98	71.82	69.08	67.49
C. suedekumii	71.94	71.85	71.92	70.60	67.29
C. casei LMG S-19264	71.91	71.99	71.84	69.12	67.87
C. pollutisoli VDS11	71.90	72.00	71.93	70.91	67.22
C. halotolerans DSM 44683	71.86	71.71	71.77	70.95	67.36
C. humireducens NBRC 106098	71.82	71.79	72.07	70.79	66.89
C. nasicanis	71.81	71.81	71.65	70.76	66.87
C. macginleyi 160811	71.79	71.65	71.58	68.62	67.84
C. vitaeruminis DSM 20294	71.76	71.71	71.51	70.42	66.74
C. hylobatis	71.73	71.67	71.57	70.59	67.06
C. lemuris	71.58	71.48	71.74	70.45	67.20

C. marinum DSM 44953	71.56	71.66	71.63	70.64	67.10
C. comes 2019	71.46	71.37	71.39	70.52	67.00
C. stationis 622DSM 20302	71.19	71.14	71.22	68.71	68.19
C. mastitidis DSM 44356	71.12	71.23	71.08	71.25	66.54
C. ammoniagenes MGYG-HGUT-01533	71.09	71.18	71.35	68.78	68.26
C. testudinoris DSM 44614	71.01	71.08	70.98	70.30	67.20
C. frankenforstense DSM 45800	70.99	70.94	70.86	71.68	66.58
C. atrinae JCM 19266	70.98	70.97	70.97	70.22	67.19
C. lehmanniae c8Ua 144	70.97	70.87	70.97	69.86	66.89
C. guangdongense DSM 107476	70.96	70.92	70.89	69.94	66.82
C. oculi NML 130210	70.93	70.93	70.51	70.56	66.82
C. pilbarense CCUG 57942	70.88	71.03	70.89	69.99	66.70
C. fournieri Marseille-P2948	70.85	70.93	71.01	69.93	66.70
C. riegelii PUDD 83A45	70.85	70.76	70.60	69.34	66.61
C. mucifaciens ATCC 700355	70.82	70.77	70.92	69.76	66.91
C. maris DSM 45190	70.81	70.81	70.74	70.19	66.84
C. ihumii	70.79	70.91	70.84	69.98	66.78
C. jeddahense DSM 45997	70.78	70.95	70.76	69.56	66.54
C. faecium	70.76	70.80	70.97	70.00	66.77
C. occultum 2039	70.76	70.76	70.71	69.86	67.26
C. gallinarum	70.75	70.69	70.71	70.08	66.80
C. gottingense DSM 103494	70.75	70.86	70.80	69.66	66.79
C. doosanense DSM 45436	70.74	70.67	70.80	69.74	66.76
C. stercoris	70.73	70.70	70.75	69.82	66.39
C. urinipleomorphum Marseille-P2799T	70.73	70.70	70.58	69.70	66.74
C. qintianiae MC1420	70.71	70.83	70.47	69.58	66.54
C. lowii NML 130206	70.68	70.82	70.58	70.61	66.97
C. imitans NCTC13015	70.68	70.75	70.63	69.75	66.88
C. sanguinis CCUG 58655	70.66	70.65	70.41	69.55	66.52
C. efficiens YS-314	70.64	70.89	70.86	70.27	66.95
C. meitnerae c8Ua 172	70.64	70.58	70.55	69.63	66.83
C. afermentans subsp l*ipophilum* CCUG 32105	70.63	70.81	70.94	70.05	66.69
C. wankanglinii zg-915	70.56	70.65	70.52	69.47	66.63
C. lujinxingii zg-917	70.54	70.55	70.64	69.67	66.77
C. uberis 18M0132	70.53	70.58	70.36	70.69	66.84
C. appendicis DSM 44531	70.49	70.44	70.45	69.86	66.77
C. mycetoides DSM 20632	70.48	70.50	70.47	69.87	66.68
C. yudongzhengii 2183	70.42	70.44	70.35	69.52	67.10
C. uropygiale JCM 32435	70.41	70.29	70.27	69.77	66.47
C. aquatimens DSM 45632	70.40	70.36	70.09	69.28	66.95

C. auris DSM 44122	70.38	70.48	70.41	69.70	66.40
C. ureicelerivorans IMMIB RIV-2301	70.37	70.46	70.54	69.44	66.58
C. genitalium ATCC 33030	70.36	70.60	70.47	69.58	66.59
C. coyleae DSM 44184	70.34	70.31	70.28	69.53	66.68
C. timonense 5401744	70.32	70.45	70.20	69.65	66.52
C. glaucum DSM 30827	70.31	70.40	70.39	69.51	66.87
C. evansiae c8Ua 174	70.29	70.28	70.04	69.41	66.57
C. pilosum NCTC11862	70.28	70.27	70.16	70.02	66.70
C. liangguodongii 2184	70.26	70.28	70.35	69.45	66.27
C. uterequi DSM 45634	70.26	70.30	70.24	69.52	66.39
C. faecale DSM 45971	70.26	70.28	70.25	69.87	66.98
C. tapiri LMG 28165	70.23	70.27	70.16	69.36	67.10
C. tuscaniense DNF00037	70.19	70.16	70.14	69.35	66.97
C. lipophiloflavum DSM 44291	70.18	70.23	70.22	69.37	66.35
C. urealyticum NCTC12011	70.17	70.30	70.03	69.56	66.35
C. lubricantis DSM 45231	70.15	69.98	70.00	69.42	67.09
C. kalinowskii 1959	70.06	70.00	69.89	69.23	66.31
C. jeikeium K411	69.97	70.10	69.90	69.36	66.75
C. hindlerae NML 93-0612	69.90	69.99	69.65	69.41	66.43
C. xerosis GS 1	69.90	69.98	69.86	69.68	65.85
C. pseudopelargi 812CH	69.90	69.93	69.80	69.35	67.45
C. aquilae dsm 44791	69.89	69.93	69.69	69.64	66.71
C. hansenii DSM 45109	69.88	69.86	69.86	69.43	65.74
C. freneyi FDAARGOS 1426	69.85	69.99	69.72	69.51	65.92
C. gerontici W8	69.84	69.71	69.80	69.35	66.91
C. pseudogenitalium CCUG 27540	69.77	69.91	69.85	69.36	66.62
C. meridianum CCM 9186	69.76	69.77	69.52	69.31	66.26
C. falsenii FDAARGOS 1493	69.75	69.78	69.93	69.37	66.81
C. macclintockiae c9Ua 112	69.73	69.96	70.13	69.51	66.96
C. zhongnanshanii zg-320	69.72	69.80	69.80	69.22	66.82
C. alimapuense VA37-3	69.70	69.65	69.63	69.15	67.38
C. propinquum FDAARGOS 1112	69.69	69.68	69.48	68.61	68.87
C. pelargi 136 3	69.68	69.73	69.60	69.42	67.12
C. antarcticum P6129	69.68	69.51	69.59	69.36	66.18
C. breve	69.66	69.78	69.72	69.10	67.09
C. pygosceleis P7374 1	69.65	69.55	69.40	69.37	66.12
C. cystitidis NCTC11863	69.64	69.71	69.55	69.12	67.04
C. megadyptis subsp *dunedinense*7B	69.64	69.82	69.82	69.21	66.73
C. deserti GIMN1.010	69.63	69.72	69.52	69.55	67.10
C. pyruviciproducens ATCC BAA-1742	69.58	69.64	69.62	68.87	65.98

C. suicordis DSM 45110	69.55	69.62	69.34	68.76	66.41
C. urogenitale LMM-1652	69.55	69.67	69.45	68.64	66.63
C. spheniscorum J11	69.53	69.85	69.74	69.44	67.64
C. atypicum R2070	69.52	69.64	69.47	70.46	66.41
C. ciconiae DSM 44920	69.50	69.67	69.74	69.01	66.66
C. capitovis DSM 44611	69.47	69.48	69.56	68.93	66.19
C. glutamicum SCgG2	69.46	69.59	69.42	69.39	66.87
C. sphenisci DSM 44792	69.45	69.39	69.36	69.02	65.84
C. otitidis ATCC 51513	69.42	69.43	69.20	69.46	65.64
C. mayonis	69.41	69.47	69.56	68.66	66.91
C. mendelii	69.41	69.47	69.56	68.66	66.91
C. renale NCTC7448	69.40	69.47	69.54	78.46	66.87
C. nuruki S6-4	69.39	69.48	69.66	69.11	65.98
C. epidermidicanis DSM 45586	69.38	69.55	69.44	69.17	66.61
C. terpenotabidum Y-11	69.35	69.42	69.24	68.92	65.98
C. argentoratense DSM 44202	69.31	69.43	69.20	69.16	66.66
C. canis CCUG 58627	69.31	69.30	69.12	68.84	66.67
C. variabile NBRC 15286	69.30	69.40	69.31	69.22	66.02
C. kalidii LD5P10	69.28	69.37	69.16	68.85	66.19
C. callunae DSM 20147	69.27	69.24	69.23	68.53	67.38
C. amycolatum FDAARGOS 1108	69.22	69.37	69.28	68.84	66.49
C. lactis RW2-5	69.20	69.36	69.25	68.85	66.07
C. heidelbergense DSM 104638	69.19	69.20	69.16	68.64	66.25
C. rouxii FRC0190	69.15	69.10	68.92	68.62	67.22
C. durum F0235	69.11	69.17	69.18	68.75	66.65
C. suranareeae N24	69.11	69.18	68.88	69.15	67.01
C. belfantii FRC0043	69.10	69.18	68.93	68.89	67.28
C. glucuronolyticum FDAARGOS 1111	69.08	69.13	68.94	68.71	65.85
C. bovis 4826	69.06	69.24	69.03	68.74	65.92
C. felinum CCUG 39943	69.02	68.98	68.97	68.91	66.93
C. ulceribovis DSM 45146	69.01	69.02	68.61	68.47	66.21
C. pseudodiphtheriticum DSM 44287	68.98	69.17	69.22	68.33	68.88
C. marambiense P5848 1	68.98	69.04	69.10	68.90	66.03
C. diphtheriae bv *mitis* str ISS 3319	68.97	69.10	69.06	68.80	67.42
C. silvaticum PO100 5	68.89	68.89	68.84	68.72	66.96
C. crudilactis JZ16	68.84	68.94	68.83	68.71	66.90
C. ulcerans 809	68.83	68.85	68.70	68.55	66.97
C. sputi DSM 45148	68.79	68.78	68.93	68.60	66.10
C. glyciniphilum AJ 3170	68.78	68.82	68.61	68.65	66.12
C. matruchotii ATCC 14266	68.73	68.81	68.57	68.16	66.66

C. neomassilliense	68.70	68.64	68.80	68.74	65.76
C. anserum 23H37-10	68.67	68.65	68.39	67.95	66.56
C. pseudotuberculosis MEX29	68.64	68.70	68.49	68.27	67.00
C. auriscanis DSM 44609	68.64	68.85	68.63	68.57	66.73
C. resistens DSM 45100	68.53	68.59	68.72	68.42	66.95
C. mustelae DSM 45274	68.22	68.16	68.08	67.78	66.77
C. kroppenstedtii DSM 44385	68.10	68.15	68.03	67.80	66.35
C. choanae 200CH	67.89	67.96	67.86	67.57	66.29
C. poyangense 4H37-19	67.48	67.61	67.53	67.48	66.88
C. freiburgense DSM 45254	67.47	67.52	67.34	67.25	66.80
C. kutscheri DSM 20755	67.36	67.41	67.20	67.38	67.27
C. caspium DSM 44850	67.28	67.34	67.01	68.33	67.01

Supplementary table S3. Virulence factors genes of *Corynebacterium* isolates from camel uterus and blood. number (1) indicate presence of virulence factor gene (0) absence of the virulence factor gene.

**Virulence factors**	**Related genes**	*Corynebacterium sp* 2581A	*Corynebacterium sp* 2583C	*Corynebacterium sp* 3274	*Corynebacterium sp* 4168A	*Corynebacterium sp* ayman	
**Adherence**							
SpaA-type pili	*spaA*	0	0	0	0	1	
SpaD-type pili	*spaD*	0	0	1	0	1	
SpaD-type pili	*srtC*	1	1	1	1	1	
SpaH-type pili	*srtE*	0	0	0	0	1	
Surface-anchored pilus proteins	*sapD*	0	0	1	0	1	
DIP0733	DIP_RS14950	1	1	1	1	1	
**Post-translational modification**							
Thioredoxin domain-containing protein [MdbA]	DIP_RS20575	1	1	1	1	1	
**Regulation**							
Diphtheria toxin repressor DtxR	*dtxR*	1	1	1	1	1	
**Nutritional/Metabolic factor**							
Iron (III) transporter	*AFuABC*	0	0	1	0	1	
Heme transporter	*hmuTUV*	1	1	0	1	1	
Iron-siderophore transporter	*fepBDGC*	1	1	0	1	1	1
Iron-siderophore transporter	*CeuABCD*	1	1	1	0	1	1
